# The role of the gut microbiota in tumor, immunity, and immunotherapy

**DOI:** 10.3389/fimmu.2024.1410928

**Published:** 2024-06-05

**Authors:** Yuyan Xie, Fang Liu

**Affiliations:** Department of Medical Oncology, Harbin Medical University Cancer Hospital, Harbin, China

**Keywords:** gut microbiota, tumors, CRC, host immunity, immune checkpoint inhibitors

## Abstract

In recent years, with the deepening understanding of the gut microbiota, it has been recognized to play a significant role in the development and progression of diseases. Particularly in gastrointestinal tumors, the gut microbiota influences tumor growth by dysbiosis, release of bacterial toxins, and modulation of host signaling pathways and immune status. Immune checkpoint inhibitors (ICIs) have greatly improved cancer treatment efficacy by enhancing immune cell responses. Current clinical and preclinical studies have demonstrated that the gut microbiota and its metabolites can enhance the effectiveness of immunotherapy. Furthermore, certain gut microbiota can serve as biomarkers for predicting immunotherapy responses. Interventions targeting the gut microbiota for the treatment of gastrointestinal diseases, especially colorectal cancer (CRC), include fecal microbiota transplantation, probiotics, prebiotics, engineered bacteria, and dietary interventions. These approaches not only improve the efficacy of ICIs but also hold promise for enhancing immunotherapy outcomes. In this review, we primarily discuss the role of the gut microbiota and its metabolites in tumors, host immunity, and immunotherapy.

## Graphical Abstract

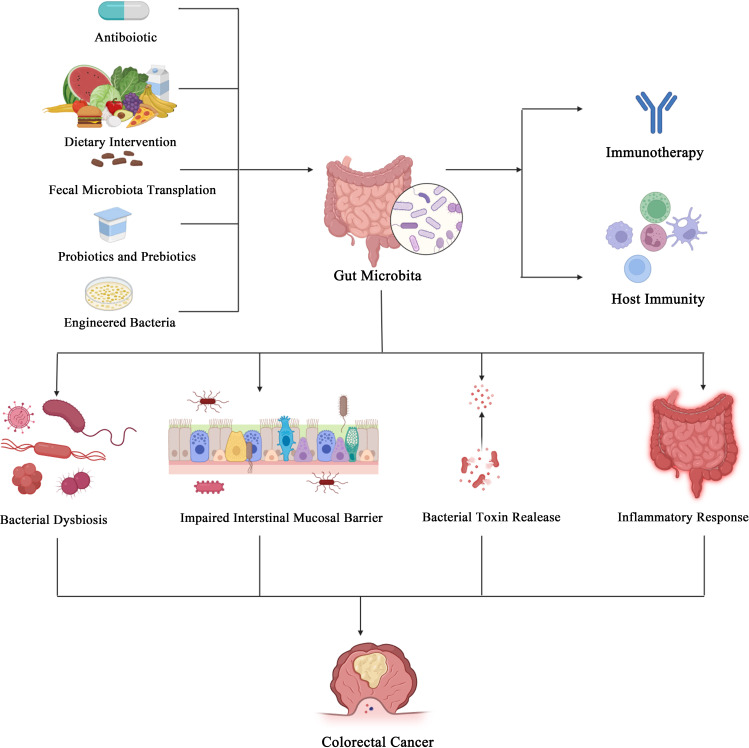

## Introduction

The collective gene content of the gut microbiota, known as the microbiome, contains approximately 3 × 10^6^ genes, which is about 150 times the length of the human genome (1). For humans, microorganisms colonize the skin, gastrointestinal tract, respiratory tract, and other sites of contact with the outside world from birth, with the gastrointestinal tract (GIT) being the most extensive interface between the organism and the outside world in direct contact (2). The human gut microbiota consists of bacteria, fungi, viruses, archaea, and potentially protozoa (3). Furthermore, the gut microbiota can contribute to the development of various diseases, including cancer. Crosstalk between gut microbiota shapes the pathology of certain cancers, such as colorectal cancer, modulating the disease at the level of predisposing conditions, initiation, genetic instability, response to comorbidities, and treatment (4). In addition, the gut microbiota promotes tumor immune activation or escape by altering the composition of the tumor microenvironment, for example, through the modulation of inflammatory cytokines or immune checkpoint molecules (5).

The gut microbiota forms the largest co-ecosystem with the host, interacting with host genes to modulate growth processes and adapt to environmental exposures. It plays a vital role in maintaining systemic immune homeostasis during bacterial infections (6) and is integral to various immune processes. The intestinal mucosal immune system consists of lymph nodes, lamina propria (which contains immune cells such as T cells and B cells), and epithelial cells, serving as a protective barrier for intestinal integrity. When the barrier is compromised, it can lead to a variety of diseases, including inflammatory bowel disease and infections. The mucosal immune system, through its various components, monitors the composition of the gut microbiota. Inflammation caused by abnormalities in the immune system affects the balance of the intestinal microbiota and can lead to intestinal-associated diseases such as Crohn’s disease and ulcerative colitis (7).

Over the past few years, ICIs have significantly improved survival in various tumors by inhibiting T-cell inhibitory receptor-ligand interactions on tumor or stromal cells and initiating T-lymphocyte-mediated immune responses (8). The current clinical use of ICIs focuses on the use of CTLA-4 antibodies (Ipilimumab), PD-1 antibodies (Pembrolizumab, Nivolumab), and PD-L1 antibodies (Atezolizumab, Durvalumab). ICIs have transformed cancer therapy and are approved for the treatment of a wide range of malignancies including, but not limited to, melanoma, renal cell carcinoma, and non-small cell lung cancer, Hodgkin’s lymphoma. In contrast to chemotherapy or targeted therapies, ICIs can induce durable responses in some patients, even after treatment discontinuation, suggesting that durable tumor-specific immune memory can be generated. In the phase III CheckMate067 trial, efficacy and safety results of up to 6.5 years were shown for Ipilimumab (NCT01844505) (9). However, due to the heterogeneity of treatment response to ICIs, only less than 30% of patients respond effectively to immunotherapy (10). In recent years, there has been growing evidence that the gut microbiota and its metabolites significantly impact ICIs by modulating both innate and adaptive immunity, such as through the regulation of T-cell function and cytokine production (11, 12), thereby influencing the efficacy of ICIs.

This paper will focus on the complex intrinsic roles of the gut microbiota and its metabolites in cancer, host antitumor immunity, and the response to immunotherapy. Additionally, we will review ongoing clinical trials that aim to manipulate the gut microbiota to enhance the efficacy of ICIs and develop therapeutic strategies to reduce adverse effects.

## Gut microbiota and tumors

### Gut microbiota and digestive tumors

#### Gut microbiota and colorectal cancer

##### Bacterial dysbiosis and colorectal cancer

Patterns of imbalance or disturbance in the gut microbiota have been recognized as indicators of specific diseases or poor health. When the delicate balance of bacteria that colonize the gut is disrupted, diversity is reduced, stability is decreased, and a more significant number of pathogenic microbiota are typically produced, leading to a variety of disease pathologic processes through adverse impacts on host immune response and function (13). In contrast, some bacteria, mainly probiotics, are depleted in CRC patients (14). Also, bacterial diversity and abundance are reduced in CRC patients (15). Yu et al. found that, unlike what was observed for bacteria, the α-diversity of fungi was not significantly different between CRC patients and healthy individuals, but the fungal community composition was significantly altered (16). The *Basidiomycota/Ascomycota* ratio was increased in CRC patients compared to healthy individuals. Malassezia spp. were enriched in CRC, whereas *Saccharomyces cerevisiae* and *Pneumocystidomycetes* were reduced (17). In addition, studies examining the accumulation of gut microbiota in experimental animal models and patients have shown that various stages of CRC disease progression are associated with significant dysbiosis in CRC tissues and microorganisms in the adjacent mucosa (18). For example, as adenomas and serrated polyps progress to CRC, toxins produced by pathogenic microorganisms tend to increase in the intestinal mucosal tissues, such as cytotoxic necrotizing factor and cycle inhibitory factor produced by Clostridium perfringens (18).

##### Impaired Intestinal mucosal barrier

The intestinal mucosal barrier serves to isolate the intestinal microbiota from immune cells, maintaining homeostasis. The intestinal mucosal barrier comprises a single layer of intestinal epithelial cell (IECs) linked by tight junctions. The mucosal barrier, composed of tight junction (TJ) proteins, serves as a defense mechanism to separate bacteria from host cells. Alterations in the epithelial membrane can increase susceptibility to infections and facilitate the transfer of microbial metabolites to the host. The gut microbiota can reduce the stability of the intestinal barrier and modify the mucosal immune system, leading to chronic inflammatory damage (19). When the physical barrier is damaged, immunogenic substances can pass through the intestinal wall and cause damage. Current studies suggest that damage to the intestinal wall can cause dysbiosis of the gut microbiota and contribute to the development of CRC through mechanisms such as induction of chronic inflammation, regulation of T cells to trigger immune responses, the release of cytotoxins, and synthesis and secretion of metabolites (20, 21). The intestinal mucosal barrier is highly permeable in human tumor and mice models. Certain pathogenic bacterial species, such as *Fusobacterium nucleatum* can stimulate an inflammatory state that disrupts the intestinal mucosal barrier through induced enteritis, leading to increased susceptibility to CRC, increased production of reactive oxygen species (ROS) (22), and altered signaling pathways to promote cancer development (23), or promote cancer by blocking the function of anti-tumor immunity (24). For example, in IL17R-expressing colonic epithelial cells, the inflammation induced by enterotoxin-producing *Pseudomonas fragilis* is initiated by a loss of intestinal barrier function, leading to rapid activation of the helper T cell 17 (TH17)-dependent inflammatory cascade response and activation of the STAT3 and NF-κB signaling pathways (25, 26). Similarly, Li et al. found that the gut microbiota of CRC patients disrupted the gut barrier, inducing low-grade inflammation and dysbiosis (27). Overall, damage to the intestinal barrier exacerbates intestinal inflammation.

##### Cascade signaling pathways

Carcinogenic signaling pathways, such as the Wnt/β-catenin, MAPK, and NF-κB cascades, are frequently activated in the cells of CRC patients, and these signaling cascades can be triggered by pathogenic bacteria (15). Many pathogens transduce their signals by directly interacting with receptors expressed on the surface of colonic epithelial cells. The bacterium *Clostridium nucleatum* expresses the cell surface adhesin FadA, which binds to E-cadherin on colonic epithelial cells and activates β-catenin signaling, leading to increased expression of cyclinD1, annexin A1 (28), Chk2 (29), and tumorigenesis. Similarly, *Porphyromonas gingivalis* (*P. gingivalis*), a significant pathogen of periodontitis, also promotes tumor progression in CRC patients. *P. gingivalis* invades cells and promotes CRC cell proliferation by activating the MAPK/ERK signaling pathway (30). In addition, Long et al. demonstrated that another *P. anaerobius*, which adheres to the oral cavity of CRC patients, drives CRC proliferation through the PCWBR2-integrin α2/β1-PI3K-Akt-NF-κB signaling axis (31). Furthermore, the bacterium *F. nucleatum* signals to MYD88 through activation of Toll-like receptor 4, leading to NF-κB activation and upregulation of miRNA-21 expression. This increases the proliferation and development of CRC cells and is associated with a poorer prognosis (32). In addition to the expression of the NF-κB pathway in epithelial cells, the regulation of this pathway by miR-148a in macrophages is also essential in CRC. In miR-148a-deficient mice, it is more likely to result in colitis and colitis-associated tumorigenesis. This is mainly because miR-148a directly targets upstream regulators of NF-κB and STAT3 signaling, leading to the activation of NF-κB and STAT3 in macrophages and connective tissues, which affects the pathogenesis of colitis and colitis-associated tumor formation (33).

##### Bacterial toxin release

**Figure 1:**
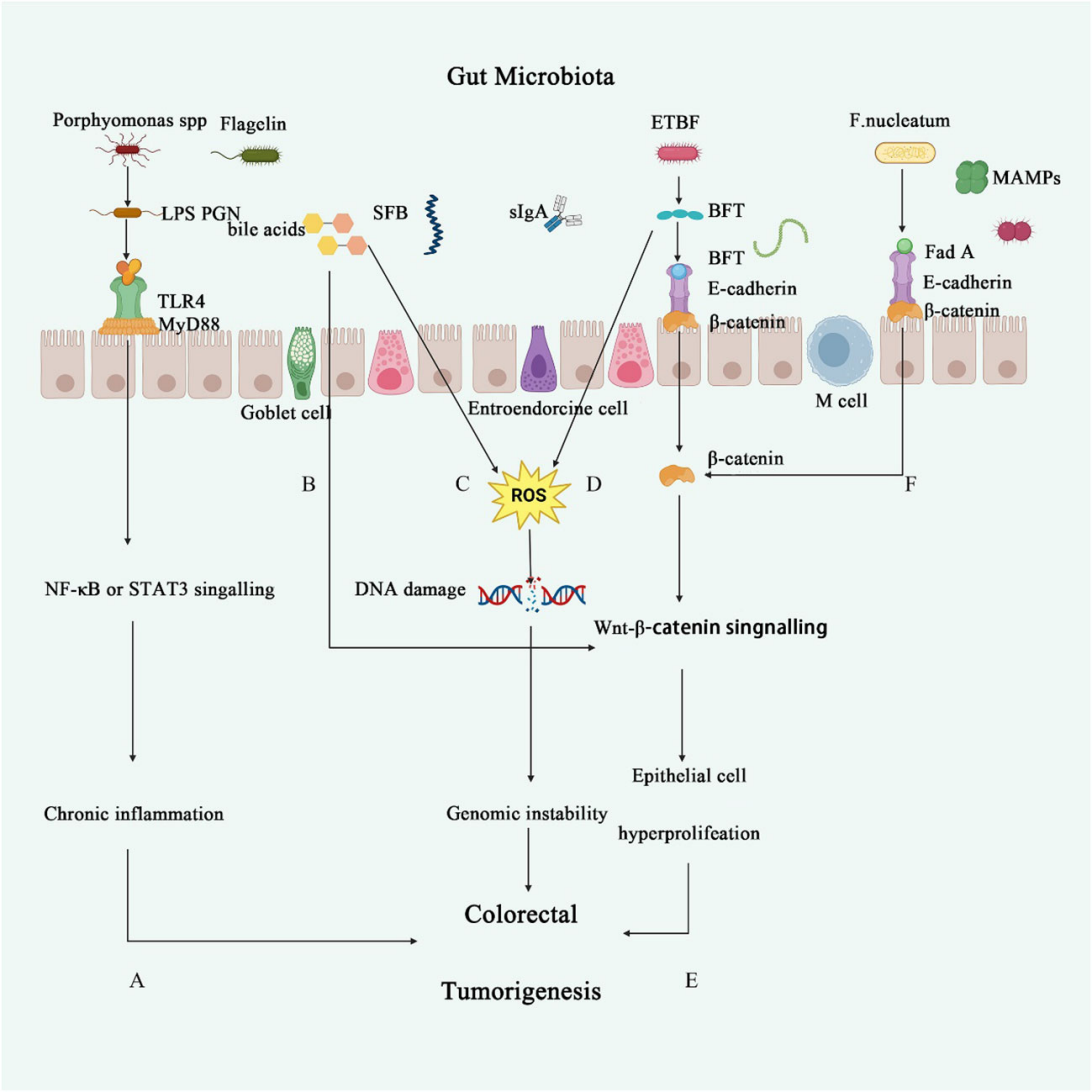
The role of intestinal microbiota in CRC. **(A)** *Porphyromonas endodontalis* and *P. gingivalis* lipopolysaccharide (LPS) induce angiogenesis through TLR2 and TLR4 activation (36). TLR4 can stimulate various inflammatory signaling pathways, such as nuclear factor-κB, interferon regulatory factor 3, and the NOD-like receptor pyrin domain containing three pathways, thereby triggering the release of pro-inflammatory markers and increasing the risk of chronic inflammation (37). **(B)** Secondary bile acids lead to elevated levels of glucans in the serum, reduce PCNA expression, and inhibit the activity of the Wnt/β-catenin pathway in intestinal cells, thereby suppressing intestinal epithelial cell proliferation and causing disruption of the intestinal barrier (38). **(C, D)** Secondary bile acids or BFT promote the production of reactive oxygen species (ROS) in inflammatory cells, leading to DNA damage and further activation of oncogenes or inactivation of tumor suppressor genes (39). **(E)** ETBF secretes BFT, which triggers an inflammatory response in intestinal epithelial cells through the interaction with E-cadherin and β-catenin, playing a crucial role in acute inflammation following ETBF infection (40). **(F)** *Fusobacterium nucleatum* significantly induces the activation of the Wnt/β-catenin signaling pathway, thereby regulating the progression of colorectal cancer (41).

Bacterial toxins play a pivotal role in the development and progression of CRC. A common characteristic of bacterial toxins is their ability to directly or indirectly cause DNA damage and alter gene expression, both of which are crucial for CRC development. Among these, *Clostridium perfringens* in the gut has been associated with CRC development. *Clostridium perfringens*, a gas-producing bacterium, may promote the precancerous lesions of CRC by activating yes-associated protein (YAP). Research has found that in the mucosal tissues of 12 patients with sessile serrated adenomas/polyps, this bacterium affects the interaction between YAP and zonula occludens-2 (ZO-2), reduces YAP phosphorylation and nuclear translocation, thereby promoting stem cell-like epithelial-mesenchymal transition, involving an increase in nuclear TEA domain family members and expression changes in various cell cycle- and adhesion-related proteins (34). *F. nucleatum* promotes tumor development by inducing inflammation and host immune responses in the CRC microenvironment. Increasing evidence suggests that *F. nucleatum* acquires potential toxicity and can destroy intestinal epithelial cells through *F. nucleatum* adhesin A (FadA) (35). Additionally, FadA can upregulate the expression of membrane-bound protein A1 through E-calmodulin, which is a regulator of Wnt/β-cadherin signaling and thus is involved in CRC progression (**Figure 1**) (28). FadA also binds to the attachment molecule VE-cadherin on endothelial cells, altering their integrity and increasing their permeability, thereby allowing bacteria to breach various barriers and enabling CRC to colonize different parts of the body (42). Several studies have shown that the enterotoxin-producing bacterium *Enterotoxigenic Bacteroides fragilis* (ETBF) produces Bacteroides fragilis toxin (BFT), which induces a robust inflammatory response and causes an ecological imbalance in the intestinal microbiota (43). A critical factor in the virulence of ETBF in CRC is BFT. The expression of BFT induces the cleavage of the extracellular domain of E-calmodulin in the colon epithelium, leading to an increase in epithelial cell permeability and activation of CRC through the β-catenin/Wnt pathway (**Figure 1**) (44). Another study found that BFT upregulates spermine oxidase (SMO) in the colonic epithelium, leading to SMO-dependent ROS production and DNA damage (45). Several studies have established a clear link between Escherichia coli (E. coli) and CRC. E. coli can carry the pathogenicity island pks, which encodes enzymes synthesizing colibactin (46). Colibactin forms DNA adducts (47) and cross-linkers (48), compounds that are thought to alkylate DNA on adenine residues, leading to double-strand breaks (**Figure 1**) (47), and producing abundant hexameric sequence motifs. Monocolonization of the APC^Min/+^ mice model with pks+ E. coli leads to colitis-associated CRC, which can be attenuated by deleting the pks pathogenicity islands (49). In addition, mammalian epithelial cells exposed to pks+ E. coli showed transient DNA damage, dysregulated DNA repair, and an increased frequency of gene mutations (50). Also, pks+ E. coli have been shown to channelize intestinal epithelial cell autophagy and DNA damage repair. Inhibition of this protective process increases the inflammatory and carcinogenic effects of E. coli in susceptible mice (50). Although these data already demonstrate that the genotoxic potential of pathogenic bacteria can alter the intestinal mucosal status, more data from CRC patients are needed to further elucidate these indirect effects.

#### Inflammatory response

Inflammation is both a hallmark characteristic and a recognized risk factor for CRC. Mouse models of colitis-associated CRC have shown that inflammation-induced changes in the gut microbiota promote CRC (51). Specifically, chronic inflammation creates favorable conditions for genome-toxic bacteria (e.g., pks+ Escherichia coli) that adhere to the colonic mucosa and induce host DNA damage, thereby promoting CRC in azoxymethane (AOM)-treated mice. Conversely, in the absence of pks+ E. coli, inflammation induction is insufficiently induced (52). Furthermore, the transplantation of feces from CRC patients into germ-free (GF) mice increases tissue inflammation and expression of pro-inflammatory genes (53). Conversely, the transplantation of fecal microbiota from long-surviving CRC patients into mice models enhances immune responses and limits tumor growth by beneficially altering the microbiota (54). Mechanistically, the gut microbiota releases chemokines that recruit immune cells to the tumor. Certain gut microbiota, such as ETBF (55), promote local tissue hyperproliferation by producing toxins that initiate inflammatory responses and recruit specific immune cell subsets. In colonic cells, BFT triggers an NF-κB and STAT3-dependent pro-inflammatory signaling cascade, leading to the production of IL-17 inflammatory cytokines (55). Pattern recognition receptors (PRRs) can also signal through NF-κB or STAT3. Toll-like receptor 4 (TLR4) is capable of sensing extracellular microbial products, lipopolysaccharides (LPS), or lipoteichoic acid from Gram-positive bacteria (32). F. nucleatum activates the TLR4 signaling pathway through its adaptor protein MYD88, subsequently activating NF-κB and the expression of downstream pro-inflammatory cytokines. Activation of NF-κB also promotes tumor growth and metastasis (32). Overall, inflammation induced by the gut microbiota can contribute to the initiation and progression of colorectal cancer (CRC).

Moreover, dysregulation of gut microbiota metabolites is also implicated in the development of CRC. In a systematic review and meta-analysis of the role of bacterial metabolite SCFAs in CRC, it was found that 70.4% of the studies reported significantly lower fecal acetate, propionate, butyrate, or total SCFAs concentrations in people at high risk for CRC, whereas in 66.7% of CRC patients, fecal acetate and butyric acid concentrations were significantly lower than those in healthy populations (56). At the genetic level, one potential initiating mechanism of CRC is the inhibition of histone deacetylation by SCFAs. This causes genetic instability and leads to an overall upregulation of genes related to SCFA signaling and chromatin regulation (57). Implantation of feces from CRC patients into an APC^min/+^ mice model revealed increased pathogenic bacterial abundance and decreased production of SCFAs by SCFA-producing bacteria and SCFAs, leading to adenoma progression (27). In addition, secondary bile acids play an essential role in the development of CRC. In patients with advanced adenomas, the concentration of porcine deoxycholic acid was significantly higher in ileal fluid, while the relative abundance of ursodeoxycholic acid was reduced (58). Analysis of bile acids in the colonic remnants or feces of patients with CRC revealed significantly higher proportions of deoxycholic acid, lithocholic acid, secondary bile acids, and the goose deoxycholic acid-lithocholic acid family (59). The secondary bile acid taurocholic acid can stimulate certain intestinal microbiota to convert taurocholic acid and other bile acids into hydrogen sulfide and deoxycholic acid, respectively, which are known genotoxins and tumor promoters (60).

#### Gut microbiota and other digestive tumors

*Helicobacter pylori* infection-induced gastric cancer is one of the typical examples of the complex relationship between the human gut microbiota and the environment. The occurrence of gastric cancer is associated with the bacterial virulence of *H. pylori*, host genetic diversity, and environmental factors (61). The oncoprotein CagA is a bacterial carcinogen composed of a structured N-terminal region and a disordered/unstructured C-terminal tail, with a molecular weight of 125–145 kDa (62). Studies on gastric epithelial cells cultured *in vitro* have shown that CagA promotes malignant transformation by imparting various oncogenic phenotypes to cells. Specifically, the tyrosine-phosphorylated CagA-induced hummingbird phenotype is mediated by the abnormal activation of the SHP2 phosphatase through SHP2-MEK-ERK signaling and SHP-FAK signaling. Additionally, non-phosphorylated intracellular CagA binds to the E-cadherin-β-catenin complex, inducing the accumulation of β-catenin, thereby promoting the transcription of oncogenes (62). On the other hand, VacA is an autotransporter and pore-forming toxin that inserts into the host cell membrane as oligomers, forming an anion channel and inducing vacuolation in epithelial cells *in vitro*. VacA primarily causes cytotoxicity by inducing or inhibiting defective autophagy, leading to the accumulation of cytotoxic substances such as ROS and p62, thereby increasing the risk of DNA mutations, genomic instability, and cancer formation (63). Moreover, VacA can also contribute to gastric epithelial damage through other pathways, such as inducing Ca^2+^ influx and ROS production, leading to the activation of NF-κB and promoting inflammatory immune responses (64). There is a synergistic effect between CagA and VacA, which enhances the trans-epithelial delivery of nutrients, providing specific nutrients such as iron to the bacteria attached to the gastric epithelium (65).

*H. pylori* has been widely recognized for its role in gastric cancer, but with the advancement of PCR technology and metagenomics, research on the relationship between the gastric microbiota and gastric cancer has gradually increased. Although the relationship between microbial diversity and gastric cancer remains inconclusive, several studies have indicated associations between specific microbiota and cancer. Dai et al. conducted 16S rRNA gene sequencing and ultra-high-performance liquid chromatography-tandem mass spectrometry on 37 gastric cancer tissues and matched non-tumor tissues, finding a higher abundance of *Lactobacillus* in gastric tumor tissues. *Lactobacillus* may play a role in the degradation and synthesis of differential metabolites, thereby promoting the occurrence and development of gastric cancer (66). Additionally, increasing research has begun to focus on the role of fungal dysbiosis in gastric cancer. High-throughput internal transcribed spacer 2 (ITS2) sequencing revealed an increased ratio of Basidiomycota to Ascomycota, a higher proportion of opportunistic fungi such as *Trichophyton* and *Malassezia*, and the loss of *Rhizopus* and *Rhodotorula* during cancer progression. Moreover, studies detected that cytokine and chemokine mRNA levels were associated with specific fungal dysbiosis (67). Whether the gastric microbiota plays a causal role in the development of gastric cancer or is secondary to changes in the gastric environment remains uncertain. However, studies have shown that regardless of *H. pylori* infection, genera such as *Lactobacillus*, *Gluconacetobacter*, *Acetobacter*, *Fusobacterium*, and *Granulicatella* are associated with gastric cancer (68). Therefore, animal and human studies are needed to verify the roles of *H. pylori* and the gastric microbiota in gastric cancer.

Emerging research has linked the gut microbiota to other digestive tumors, including hepatocellular carcinoma (HCC) and pancreatic cancer (PDAC). Through the portal system, the liver is uniquely exposed to intestinal bacterial components and their metabolites, which may result in inflammatory changes and hepatotoxicity, leading to cancer development. In patients with HCC, the numbers of *Bacteroidetes* and *Agrobacterium tumefaciens* are increased, and the number of *Bifidobacterium* is decreased. *Bacteroides akermanii* and *Bifidobacterium bifidum* were negatively correlated with calreticulin concentrations, which correlated with humoral and cellular markers of inflammation (69). The formation of non-alcoholic fatty liver disease-associated hepatocellular carcinoma (NAFLD-HCC), which can be induced by cholesterol, has been associated with dysregulation of the gut microbiota. *Mucispirillum, Desulfovibrio, Anaerotruncus*, and *Desulfovibrionaceae* were sequentially increased in NAFLD-HCC mice models, whereas *Bifidobacterium* and *Bacteroides* were decreased in high-fat/high-cholesterol (NAFLD) -fed mice (70). In addition, the enterohepatic recycling of deoxycholic acid (DCA) can stimulate hepatic stellate cells (HSCs) to adopt a senescence-associated secretory phenotype (SASP), which in turn secretes a variety of inflammatory and tumor-promoting factors in the liver, thereby promoting the development of HCC in mice after exposure to chemical carcinogens (71). In addition, it has been shown that alterations in bile acids can affect hepatic metabolic homeostasis and contribute to the development of HCC. Ma et al. found a link between bile acid metabolism controlled by intestinal bacteria and hepatic antitumor immunity. Altered commensal gut bacteria in mice induced selective antitumor effects in the liver, with an increase in hepatic CXCR6^+^ natural killer T (NKT) cells and increased interferon γ production after antigenic stimulation (72). Further clinical studies are needed to determine whether there is a correlation between specific gut microbiota and HCC and whether the changes seen are associated with disease progression.

Historically, the pancreas was thought to be sterile, but recent evidence has revealed the presence of intra-tumor microbes and their impact on pancreatic cancer progression and treatment efficacy (54). Using 16S rRNA gene sequencing, high alpha diversity was found in the tumor microbiome of long-term surviving PDAC patients. In addition, an intra-tumor microbiome marker (*Pseudoxanthomonas-Streptomyces-Saccharopolyspora-Bacillus clausii*) was identified, and this biome was found to be associated with long-term survival (54). Aykut et al. found that excision of the fungal community in a model of slow-progressing and invasive PDAC had a protective effect on tumor growth. In contrast, the repopulation of species of the genus *Marathonella* accelerated tumorigenesis (73). Trimethylamine N-oxide (TMAO), a metabolite derived from the gut microbiota, enhances antitumor immunity against PDAC. In a mice model of PDAC, the combination of TMAO with ICIs significantly reduced tumor load and improved survival; TMAO enhanced the type I interferon (IFN) pathway and conferred antitumor effects in a type I IFN-dependent manner (74). Furthermore, in a colon cancer model, Geller et al. found that bacteria can metabolize gemcitabine (2’,2’-difluorodeoxycytidine), a drug used to treat PDAC, into its inactive form (75). The crosstalk between these organs suggests a potential presence and influence of the gut microbiota in distal digestive system organs, warranting further elucidation of the specific sources and pathogenic mechanisms.

### Gut microbiota and non-digestive tumors

The gut microbiota not only affects digestive system tumors but also impacts tumors in other sites. A specific study found that the gut microbiota is directly involved in the efficacy of trastuzumab treatment (75). Patients who achieved complete remission (CR) exhibited lower alpha diversity and abundance of *Lachnospiraceae, Turicibacteraceae, Bifidobacteriaceae*, and *Prevotellaceae*, similar to antibiotic-treated mice (75). Dubigeon et al. collected feces from breast cancer patients and healthy women for microbial comparisons and found that microbiota diversity was reduced in breast cancer patients, with a relative enrichment in Firmicutes and a depletion in Bacteroidetes compared to those of healthy women. A tendency towards a decreased relative abundance of *Odoribacter* spp.*, Butyricimonas* spp.*, and Coprococcus* spp. was observed (76). In addition, Zhu et al. found that in postmenopausal women with breast cancer, the intestinal macrogenome was enriched with genes encoding lipopolysaccharide biosynthesis, iron complex transporter system, PTS system, secretion system, and β-oxidation (77).

An imbalance of microbiota in the respiratory and intestinal tracts, known as ecological dysbiosis, is associated with alterations in immune responses and the development of lung disease (78). Fecal microbiota analysis showed that non-small cell lung cancer patients were associated with significant upregulation of *Prevotella, Gemmiger*, and *Roseburia* when gut microbiota was dysbiotic (79). By examining the role of gut microbiota in cachexia in 31 lung cancer patients, it was found that in patients with cachexia, the gut microbiota showed increased consumption of branched-chain amino acids (BCAAs) and methylhistamine, with alterations in microorganisms involved in functional pathways (80). Analysis of the gut microbiota in lung cancer by 16S rRNA gene sequencing revealed specific microbiota. Firmicutes and Actinobacteria were significantly reduced, whereas *Aspergillus, Ruminococcus* spp., uncharacteristic genera of *Enterobacteriaceae*, and uncharacteristic genera of *Lactobacillaceae* showed high abundance (81). In patients with advanced NSCLC treated with first-line ICIs, it was found that those with an objective response rate (ORR) and progression-free survival (PFS) > 6 months had an enrichment of *Gastrocysticercaceae* UCG-013 and *Agathobacter*, while patients with overall survival (OS) > 12 months were enriched in *Gastrocysticercaceae* UCG-013 (82).

## Gut microbiota metabolite

Short-chain fatty acids (SCFAs) are metabolites produced by the fermentation of insoluble dietary fiber by gut microbiota. They directly activate G protein-coupled receptors (GPCRs) and inhibit histone deacetylases (HDACs), acting as an energy link between the gut microbiota and dietary patterns, thereby contributing to improved gut health (83). The main SCFAs include butyrate, propionate, and acetate. Among them, butyrate is particularly important for maintaining colonic health as it is the primary energy source for colonic epithelial cells. SCFAs are rapidly absorbed by colonic cells through various pathways, including diffusion in their undissociated form and active transport mediated by monocarboxylate transporter 1 (MCT1) or sodium-coupled monocarboxylate transporter 1 (SMCT1) in their dissociated form. Once absorbed, they participate in the mitochondrial citric acid cycle, generating more adenosine triphosphate (ATP) for cellular metabolism (84). Additionally, SCFAs regulate T-cell metabolism by inhibiting HDACs, promoting the differentiation of naive T cells into Th1 and Th17 effector T cells, which is likely related to HDAC inhibition activity (85). Furthermore, SCFAs maintain homeostasis by regulating related signaling cascades through binding to several GPCRs, particularly GPR43, GPR41, and GPR109A (86).

Bile acids, synthesized in the liver, possess direct or indirect antimicrobial properties and can regulate the composition of the microbiota, thereby influencing the size and composition of the bile acid pool (87). Studies have found that the administration of bile acids can alter both the bile acid pool and the composition of the gut microbiota. For example, in Apc^min/+^ mice, cholic acid increases the prevalence of opportunistic pathogens such as *Prevotella* and *Desulfovibrio* while reducing beneficial bacteria like *Clostridium*, *Lactobacillus*, and *Roseburia (* 88). Additionally, compared to control groups, mice fed a diet supplemented with deoxycholic acid exhibited a significant increase in the numbers of Bacteroides and Parabacteroides, while the numbers of *Lactobacillus*, *Clostridium clusters*, and *Clostridium cluster XIV* decreased (89). Dietary (90) changes can lead to alterations in the gut microbiota, subsequently affecting the host’s metabolic phenotype and disease risk. Many bile acids regulated by bacteria serve as key signaling molecules that fine-tune host metabolism. Disruption of bacterial communities and the resultant changes in the size and composition of the bile acid pool can lead to metabolic and immune dysregulation (91).

It should be noted that the impact of gut microbiota metabolites is complex and diverse, and there are still many unresolved questions regarding their mechanisms of action in tumorigenesis and immune regulation. The effects of different metabolites may vary depending on changes in the microbial composition. Therefore, further research is needed to understand the role of gut microbiota metabolites in the body, which can aid in the development of novel strategies for cancer prevention and treatment.

## Interactions between immune system and the gut microbiota

Host immunity regulates the gut microbiota to maintain a stable internal environment, while the microbiota, in turn, influences host immunity. Disturbances in the gut microbiota and disruption of gut mucosal integrity can lead to gut dysbiosis, contributing to immune-related disorders such as inflammatory bowel disease and cancer. The gut microbiota can influence intestinal immunity and modulate immune responses at distal mucosal sites through mechanisms involving circulation, systemic metabolism, and immune regulation (92). Increasing evidence of molecular mechanisms suggests that the gut microbiota plays a critical role in modulating host immunity in both health and cancer. Overall, it is essential to discuss recent advances in understanding the role of the gut microbiota in shaping the immune system.

### Innate immunity and the gut microbiota

The innate immune response plays a vital role in maintaining the homeostasis of the internal environment by clearing pathogenic bacteria and modulating adaptive responses to the microbiota. These effects are mediated through sIgA (93), TLR4 (94), autophagy (95), and inflammatory vesicles (95). sIgA, the primary immunoglobulin in the gut, targets polysaccharides and flagellin on the surface of bacteria, leading to the formation of sIgA-encapsulated bacteria. sIgA-encapsulated Lactobacillus rhamnosus contributes to the growth of Dendritic cells(DCs), particularly in Peyer’s patches. In addition, sIgA-encapsulated *L. rhamnosus* upregulates Toll-like receptor 2 (TLR2) expression in 3-week-old mice (96). LPS, a significant component of the outer membrane of Gram-negative bacteria, bind to the TLR4-MD2 complex to activate innate immunity (97). Pro-inflammatory hepatic macrophages produce ROS through phagocytosis of monomeric TLR4-MD2 complexes by NADPH oxidase 2. Palmitate-stimulated CD11b+F4/80low hepatic macrophages produce ROS through initiator protein-mediated endocytosis of TLR4 and NOX2, ROS production, and increased expression of IL-1β in macrophages (98). Inflammasomes, also known as inflammatory vesicles, are cytoplasmic protein complexes that play a crucial role in inflammation and cell death. Inflammasomes can be activated by a variety of pathogen-associated molecular patterns (PAMPs) and damage-associated molecular patterns (DAMPs), and activation of inflammasome motifs by PAMPs and DAMPs leads to the activation of cysteinyl asparagine-2016, which cleaves the precursors of IL-1β and IL-1 in order to produce the mature forms of these cytokines (99, 100). In addition, NLRP12 plays an essential role in inflammatory vesicles, inducing inflammatory vesicle-dependent release of IL-1β and IL-18 during Yersinia pestis or Plasmodium falciparum infection. NLRP12 acts as a negative regulator of NFκB and MAPK signaling pathways during infection with *Salmonella enterica serotype S. Typhimurium, vesicular stomatitis virus, Mycobacterium kansasii*, or *Mycobacterium tuberculosis*, as well as colon tumorigenesis (101).

In homeostasis, myeloid cells represent the most abundant immune cell type in the digestive system, among which DCs and macrophages play crucial roles in maintaining epithelial barrier integrity and anti-tumor immunity. For instance, DCs expressing FFAR2 inhibit their expression of IL-27 to sustain murine intestinal mucosal barrier integrity, reduce tumor bacterial burden, and suppress CD8^+^ T cell exhaustion, thereby restraining tumor initiation (102). DCs from gut-associated lymphoid tissues, spleen, and tumor-draining lymph nodes sense and respond to specific bacteria, stimulating anti-tumor immunity by producing IL-12, thereby amplifying anti-tumor T cell responses (103)and/or IFN-mediated (104) signaling. Intestinal macrophages promote gut barrier integrity and restrict tumor development. In patients with inflammatory bowel disease and colorectal cancer associated with colitis, intestinal macrophages respond to dysbiotic microbiota and metabolites that disrupt the epithelium. For example, inherent FXR receptors in intestinal macrophages perceive aberrant bile acids, leading to the release of pro-inflammatory factors, thereby promoting intestinal stem cell proliferation (105). The gut microbiota stimulates monocyte-derived macrophages to produce IL-1β upon exposure to lipopolysaccharides, inducing T helper cell production of interleukin 17, thus creating an inflammatory environment conducive to tumor initiation (106).

Abnormal communication between the innate immune system and the gut microbiota may lead to complex diseases. A study identified that the gut microbiota plays an essential role in modulating peripheral cGAS-STING activation, thereby promoting systemic resistance to viral infections (101). Complete loss of the innate immune signaling molecule TAK1 in myeloid cells (Tak1ΔM/ΔM) leads to complete resistance to chemically induced colitis and CRC, a phenomenon associated with microbiome alterations that drive protective immunity (107). Analysis of MyD88/TRIF knockout mice with nuclear ATF6 expression in intestinal epithelial cells (nATF6IEC) showed that tumor development was dependent on the activation of STAT3 through MyD88/TRIF signaling (108). In a model of DSS-induced colitis, the PI3K/PTEN signaling pathway in DCs was found to enhance IL-6 production, and DC-specific PTEN knockout (PTENΔDC) resulted in heightened Th1 cell responses and increased mortality (109). Wang et al. performed RNA sequencing analyses to show that simulated microgravity (SMG) inhibits the production of inflammatory cytokines, including TNF-α and IL-6, and suppresses the activation of innate immune signaling in enteropathogenic Escherichia coli (EPEC)-infected macrophages through the p38MAPK and Erk1/2MAPK pathways (110).

Gut microbiota metabolites play a significant role in immunomodulation. SCFAs, produced by microbial fermentation of dietary fibers, have been shown to induce the differentiation of regulatory T (Treg) cells *in vitro* and *in vivo*, and mitigate colitis development induced by the transfer of CD4(+) CD45RB(hi) T cells in Rag1(-/-) mice (111). Specifically, propionate-derived SCFAs promote the expansion of colonic Tregs by inhibiting histone deacetylase (HDAC) activity and targeting forkhead box protein P3 (FOXP3), a key nuclear transcription factor of Tregs (111). SCFAs also upregulate the production of interleukin-22 (IL-22) by promoting the expression of the aromatic hydrocarbon receptor (AhR) and hypoxia-inducible factor 1α (HIF1α), which enhances intestinal homeostasis (112). Bile acids are also crucial in innate immunity. The agonist INT-747, a Farnesoid X receptor agonist, significantly reduces TNF-α secretion in activated human peripheral blood monocytes, purified CD14 monocytes and DCs, and monocytes from the lamina propria of inflammatory bowel disease (IBD) patients (113).

### Adaptive immunity and the gut microbiota

The mammalian gut microbiota plays a crucial role in the development and maturation of the host immune system, particularly during the adaptive immune response. Regulatory T (Treg) cells and helper T (Th17) cells are the most abundant subpopulations of lamina propria CD4^+^ T cells under steady-state conditions (114). Th17 cell cytokines are important activators of the innate immune system (115). Treg cells are vital for developing oral tolerance and suppressing hyperinflammatory responses to numerous resident commensal bacteria. The balance between Treg cells and Th17 cells is crucial for mucosal immune homeostasis. Certain Clostridium spp. clusters, such as IV and XIVa, promote the accumulation of Treg cells in the colonic mucosa (116). ETBF promotes the differentiation of preneoplastic mononuclear cells and myeloid-derived suppressor cells (MO-MDSCs) through the combined action of its toxins BFT and interleukin-17 (IL-17) on the colonic epithelium. ETBF selectively upregulates nitric oxide synthase 2 (NOS2) and arginase 1 (ARG1), leading to nitric oxide (NO) production and inhibition of T cell proliferation (117). Segmented filamentous bacteria (SFB) are symbionts that drive postnatal maturation of the intestinal adaptive immune response. SFB stimulate isolated lymphoid follicles and tertiary lymphoid tissues, acting as an alternative to Peyer’s patches for inducing intestinal Th17 cell responses (118). Analysis of the microbiota in immunodeficient Rag1(-/-) mice compared to wild-type mice using 16S rRNA gene sequencing revealed a high enrichment of the mucin-degrading bacterium *Akkermansia muciniphila (A.muciniphila)* in Rag1(-/-) mice (119).

**Figure 2:**
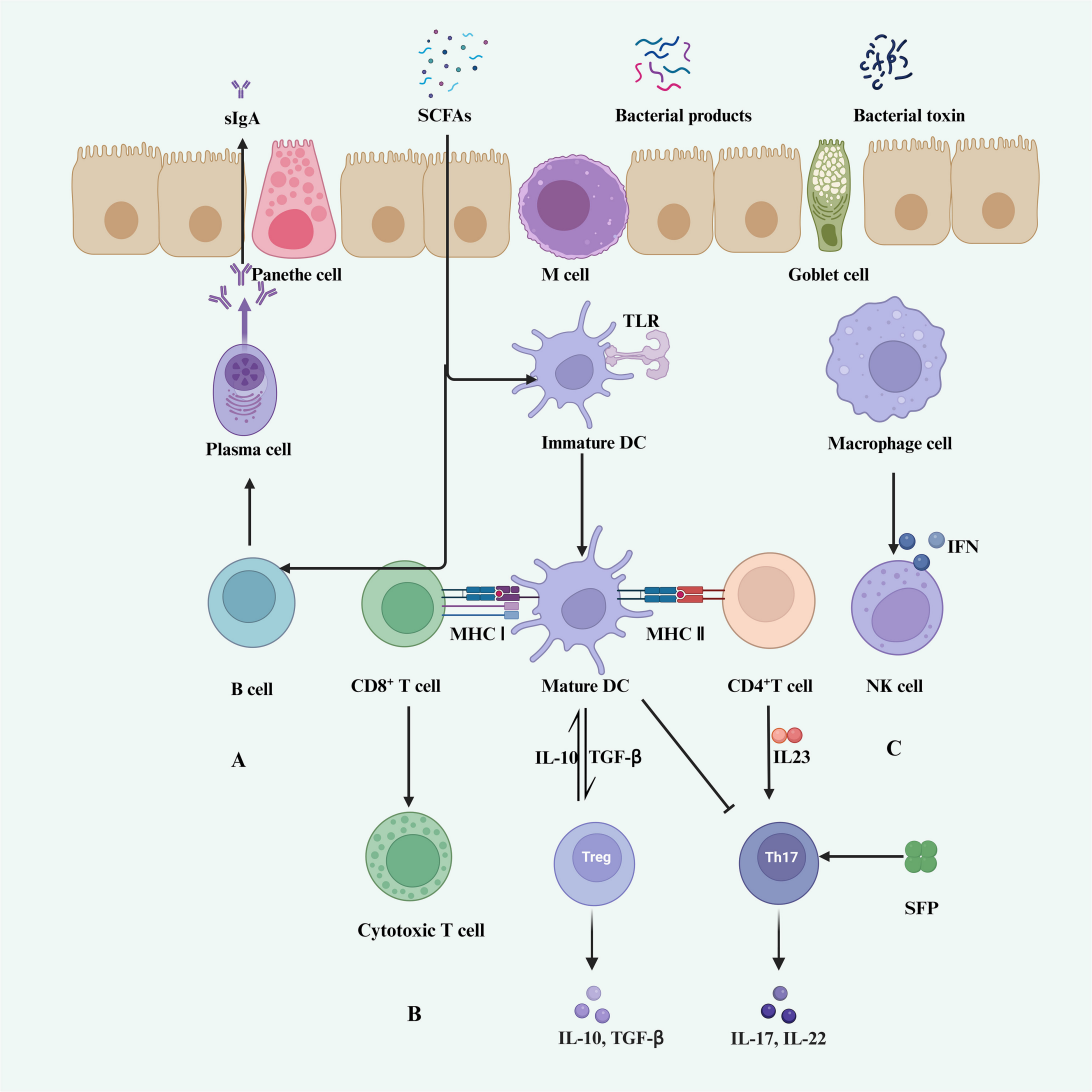
Gut microbiota and host immunity. **(A)** SCFAs promote B cell differentiation into germinal center B cells and plasma cells, stimulating the secretion of sIgA in lymphoid tissues (120). **(B)** DCs and macrophages are regulated by SCFAs, indirectly modulating T cell activity. **(C)** Immature DCs differentiate into mature DCs through recognition of TLRs, thereby promoting the activation of CD8^+^ T cells and CD4^+^ T cells. CD8^+^ T cells differentiate into cytotoxic T cells, while mature DCs promote the secretion of IL-10 and TGF-β by Treg cells. CD4^+^ T cells stimulate the secretion of IL-17 and IL-22 by Th17 cells. **(C)** Macrophages activate NK cells through IFN stimulation.

Several studies have shown that specific gut microbiota can induce the presence of CD8^+^ T cells in the systemic circulation or tumor microenvironment (TME). Modulation of the gut microbiota, as observed in preclinical and preliminary clinical studies, is associated with increased infiltration of CD8^+^ effector T cells into tumors. This infiltration is typically accompanied by enhanced activity of type 1 helper T cells and dendritic cells within the tumor and a decrease in the density of immunosuppressive cells (**Figure 2**) (121). Tanoue et al. identified 11 bacterial strains from healthy human feces that could strongly induce IFN-γ-producing CD8^+^ T cells in the gut. Additionally, colonization of mice with a mixture of these 11 strains enhanced the host’s resistance to Listeria infection and improved the therapeutic effects of ICIs (103).

An increase in CD8^+^IFNγ^+^ T cells has been associated with intestinal tumorigenesis. Dysbiosis of the intestinal flora can promote chronic inflammation and early T-cell depletion by overstimulating CD8^+^ T cells, which reduces anti-tumor immunity and increases susceptibility to colon tumors (122). In mice infected with the influenza virus, disruption of the intestinal microbiota induced by gentamicin leads to elevated levels of branched-chain amino acids (BCAAs). This inhibits the development of CD11b^+^Ly6G^+^ cells and results in overactive CD8^+^ T cell responses, thereby exacerbating the severity of viral infection (123).

SCFAs regulate colonic Treg cell homeostasis. Treatment of initial CD4^+^ T cells from healthy donors with SCFAs increased the frequency of CD4^+^CD25^+^Foxp3^+^ cells and, to a lesser extent, the proliferation of differentiated Treg cells (124). SCFAs also regulate the size and function of the colonic Treg pool and prevent colitis in mice in a *Ffar2*(GPR43)-dependent manner (125). During the active immune response, SCFAs promote the production of Th1 and Th17 cells. In T cells, inhibition of HDAC by SCFAs increases the acetylation of p70S6 kinase and phosphorylation of rS6, regulating the mTOR pathway required for the production of Th1, Th17, and IL-10(+) T cells (126). Liang et al. demonstrated differential regulation of Th1 and Th17 cell differentiation by butyrate, which also promoted IL-10 production to control T-cell-induced colitis when butyrate-treated CBirT cells were transferred into Rag1-/- mice (127). SCFAs profoundly affect the mTOR pathway in T cells and cellular metabolism. Oral administration of butyrate, an SCFA, improved humoral immunity by promoting Akt-mTOR-promoted plasma cell production in vancomycin-treated mice. Valeric acid lipids and butyrate lipids led to increased production of effector molecules, such as CD25, IFNγ, and TNFα, through metabolic and epigenetic reprogramming, significantly enhancing anti-tumor activity of specific CTL and transgenic (CAR) T cells in murine melanoma and pancreatic cancer models (128).

### Gut microbiota in the tumor microenvironment

As a significant factor influencing the TME, the gut microbiota impacts tumor progression through its metabolites, genotoxins, and signaling pathways. In an APC ^Min/+^ mice model of intestinal tumorigenesis, *Clostridium nucleatum* increased tumor diversity and selectively recruited myeloid-derived suppressor cells (MDSCs), thereby promoting tumor progression (129). An experiment analyzing the relationship between CD8^+^ T cells and melanoma found that the intestinal microbiota within the tumor modulates chemokine levels, influencing the infiltration of CD8^+^ T cells and impacting the survival of melanoma patients (130). In a mice model of primary gastric cancer established by Peng et al., methyl bacillus significantly reduced the diversity of tumor microbiota and the expression of transforming growth factor beta (TGFβ) and CD8^+^ tissue-resident memory (TRM) cells in the tumor microenvironment of gastric cancer (131). IL-25 induced alternate macrophage activation in the TME, promoting HCC cell migration, invasion, and tumorigenesis *in vivo* and *in vitro*. This induction increased the expression of waveform protein, Snail, and phosphorylated ERK, and decreased the expression of E-cadherin in HCC cells (132). In CRC, B*ifidobacterium adolescentis* induced a new subset of CD143+ cancer-associated fibroblasts (CAFs) to suppress tumorigenesis through the Wnt signaling-regulated gene, GAS1, as revealed by single-cell RNA sequencing (133). In antibiotic-treated or GF mice, tumor-infiltrating bone marrow-derived cells showed poor response to treatment, resulting in reduced cytokine production and tumor necrosis after CpG oligonucleotide treatment, and insufficient ROS and cytotoxicity production after chemotherapy (134). Additionally, single-cell RNA sequencing has shown that a lack of microbiota predisposes the TME to tumorigenic macrophages. Mechanistically, microbiota-derived stimulators of interferon genes (STING) agonists induce production of interferon I (IFN I) by monocytes within the tumor, modulating macrophage polarization and natural killer (NK) cell- DCs crosstalk (135). Researchers have found that the STING agonist cdAMP induces the production of IFN-I, which can increase the number and functionality of tumor-infiltrating NK cells and DCs, thereby enhancing the overall anti-tumor immune response. This indicates that the microbiota modulates DC-NK cell crosstalk within the TME through the STING-IFN-I signaling pathway, playing a crucial role in anti-tumor immunity (135). These biological targets offer new avenues for future interventions using the gut microbiota to manage tumor development, treatment, and prognosis.

## Gut microbiota and immunotherapy

**Figure 3:**
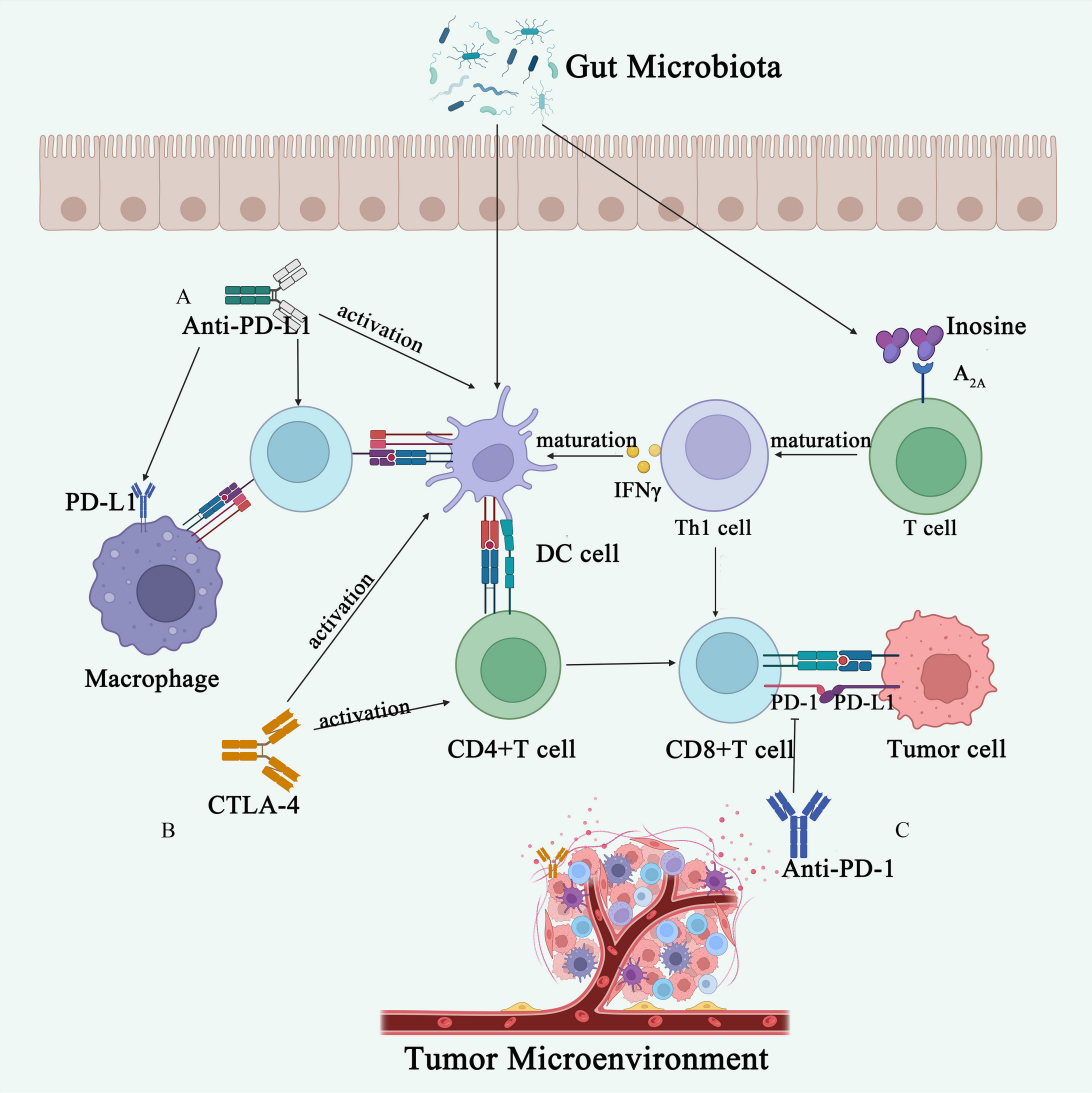
Gut microbiota influences immunotherapy. **(A)** The anti-PD-L1 antibody activates DCs, thereby enhancing the immune response ability of CD8^+^ T cells. The gut microbiota metabolite adenosine binds to the A_2A_ receptor on T cells, promoting the maturation of Th1 cells, increasing IFN-γ levels, and consequently enhancing the anti-tumor immune response in the body. **(B)** CTLA-4 inhibitors activate DCs and CD4^+^ T cells, promoting anti-tumor immunity. **(C)** Anti-PD-1 antibodies act on the surface of T cells, inhibiting tumor immune evasion.

The effect of gut microbiota on response to ICIs was initially demonstrated in a mouse model, with a landmark article published in Science in 2015 highlighting the influence of gut microbiota composition on response to CTLA-4 (136) and PD-L1 (137) inhibitors. In mice receiving anti-CTLA-4 treatment, the gut microbiota composition changed significantly, showing a relative increase in *Bacteroidetes* and *Burkholderia* and a decrease in Clostridium. Efficacy of anti-CTLA-4 treatment was notably reduced in GF mice and specific pathogen-free (SPF) mice treated with broad-spectrum antibiotics. Moreover, oral administration of *Mycobacterium fragilis* along with *Bacteroides thetaiotaomicron* or *Burkholderia cepacia* enhanced the effects of anti-CTLA-4 therapy by stimulating Th1 responses in lymph nodes and promoting intratumoral dendritic cell maturation (136) (**Figure 3**). A global prospective study involving 39 melanoma patients receiving CTLA-4 (Ipilimumab) in combination with anti-PD-1 therapy (Nivolumab) or PD-1 therapy only, identified enrichment of filamentous bacilli of the genus *Bacterium hordemannii*, anaplastic bacilli of the species *Polyporus*, and *Clostridium pumilus* in responders to the combination therapy. Formate-producing bacilli were enriched in responders to PD-1 therapy alone (138). Another large cohort study including patients with renal cell carcinoma, NSCLC, and uroepithelial carcinoma, found that patients with higher abundance of *Ackermannia* responded better to PD-1 therapy (12). The study also noted significant differences in microbial diversity and composition of fecal samples between responders and non-responders to ICIs. Fecal microbiota transplantation (FMT) from responders into antibiotic-treated/GF mice enhanced the antitumor effects of PD-1 inhibitors, while FMT from non-responders did not. Oral administration of *Akkermansia* to non-responders enhanced the efficacy of PD-1 inhibitors, indicating a potential role for the gut microbiota in mediating clinical responses to ICIs (12).

Gut microbiota has been strongly linked to the response to ICIs, although the precise mechanisms remain unclear. Studies suggest that different species of Bacteroides may play a crucial role in the antitumor effects of ICIs. GF mice and mice treated with antibiotics did not respond to anti-CTLA-4 treatment (12). However, administration of Bacteroides fragilis in these mice induced Th1 immune responses in lymph nodes and promoted the maturation of intratumoral DCs, thus restoring their response to CTLA-4 blockade (12). Similar findings in patients indicate that the efficacy of CTLA-4 antibodies is associated with T-cell responses mediated by *Bifidobacterium fragilis* or *Lactobacillus* polymorphus (139). Moreover, *Bifidobacterium shortum*, *Bifidobacterium longum*, and several other strains have been reported to enhance DC function, leading to the initiation and accumulation of CD8^+^ T cells in the TME. Supplementation with these strains may enhance the therapeutic efficacy of ICIs in cancer patients (**Figure 3**). Additionally, certain bacteria have been found to inhibit melanoma growth by attenuating the unfolded protein response (UPR) signaling pathway and mediating antitumor immunity (103, 140).

In mice modeling trials, most studies have focused on the adaptive immunity induced by the gut microbiota during treatment with ICIs. One possible mechanism is that the gut microbiota promotes anti-tumor CD8^+^ T-cell responses during ICI treatment. Zhu et al. found that serum butyrate levels in non-small cell lung cancer (NSCLC) patients were positively correlated with the expression of PD-1 on circulating CD8^+^ and Vγ9 Vδ2 (Vδ2+) T cells (141). Mechanistically, butyrate increased histone 3 lysine 27 acetylation (H3K27ac) in the promoter region of Pdcd1 and Cd28 in human CD8^+^ T cells, thereby promoting PD-1/CD28 expression and enhancing the efficacy of PD-1 therapy (141). Furthermore, pectin significantly enhanced the anti-PD-1mAb efficacy in humanized tumor-bearing mice with gut microbiota from CRC patients. 16S rRNA gene sequencing demonstrated that pectin significantly increased gut microbial diversity and beneficially modulated microbial composition. In addition, butyrate-producing bacteria were significantly enriched in the anti-PD-1mAb + pectin group, suggesting a favorable response to immunotherapy (142). Among 32 NSCLC patients, the abundance of *E. hirae* strain 13144 was found to be higher in the responder (R) group compared to the non-responder (NR) group. This increase was associated with enhanced peripheral blood CD8^+^ or CD4^+^ T-cell responses, increased IFNγ production, and significantly prolonged PFS in the R group (12). Najafi et al. observed that *Salmonella* and *Porphyromonas gingivalis* may promote cancer progression by up-regulating PD-L1 expression (143). This suggests that the intestinal microbiota could directly influence PD-L1 expression, potentially impacting the response to cancer immunotherapy (143). Collectively, these findings suggest that the gut microbiota of cancer patients may modulate immune cell responses, thereby influencing the effectiveness of anticancer immunotherapy.

### Gut microbiota as a prognostic biomarker for immunotherapy

Recently, numerous studies have highlighted the potential of the gut microbiota as a prognostic biomarker for predicting responses to ICIs. In patients with advanced gastrointestinal (GI) cancers treated with ICIs, analysis of fecal samples by Peng et al. revealed an elevated *Prevotella/Bacteroides* ratio post-treatment, correlating with a more favorable response to ICIs (144). Similarly, patients with unresectable hepatocellular carcinoma undergoing ICI therapy showed significant enrichment of *Lachnoclostridium* and *Prevotella 9* in their fecal microbiota (145). For mCRC and NSCLC patients receiving Cetuximab + Avelumab, *Agathobacter* and *Blautia* strains were identified as potentially crucial factors associated with anti-tumor activity, indicating the potential of gut microbiota as a prognostic biomarker (146). In metastatic melanoma patients treated with avelumab, higher levels of *Faecalibacterium* and other butyrate-producing bacteria were linked not only to prolonged survival but also to increased avelumab-induced colitis (147). Additionally, intestinal *A.muciniphila* has emerged as a potential prognostic biomarker for PD-1 inhibitors in mNSCLC patients, showing associations with improved ORR and OS, and exhibiting a predictive value superior to PD-L1 expression in a multivariate analysis (148).

Pathogenic bacteria, such as *F. nucleatum*, have emerged as potential diagnostic or prognostic markers. Genomic data from cancer patients have demonstrated an enrichment of *F. nucleatum* in CRC tissues compared to normal tissues. *F. nucleatum* levels have been observed to increase with tumor malignancy and are associated with metastasis. Interestingly, higher levels of *F. nucleatum* have been linked to an improved response to PD-1 inhibitor therapy in CRC patients. Additionally, *F. nucleatum* has been shown to enhance the anti-tumor effects of PD-L1 inhibitors in CRC mice models, leading to prolonged survival. Mechanistically, *F. nucleatum* induces PD-L1 expression through activation of the STING signaling pathway, leading to increased accumulation of IFN-γ^+^CD8^+^ tumor-infiltrating lymphocytes (TILs) during PD-L1 blockade therapy, thus enhancing tumor sensitivity to PD-L1 blockade. Furthermore, patient-derived organoid models have corroborated these findings, showing that higher *F. nucleatum* levels are associated with an improved therapeutic response to PD-L1 blockade (149). However, the relationship between *F. nucleatum* and tumor methylation status, which is crucial for its role as a prognostic marker, requires further clarification.

Given that various members of the intestinal microbiota can produce microbial metabolites and structures associated with the efficacy of ICIs, analyzing the functional capacity of the gut microbiota offers a promising approach to identifying predictive markers of ICI responsiveness. For instance, certain phages such as *A. muciniphila*, as well as specific bacterial species like *B. intestinihominis* and *B. thetaiotaomicron*, have been mechanistically linked to improved therapeutic responses through immunomodulation, highlighting their potential translational significance (12, 150, 151). However, a major challenge lies in achieving consensus across studies regarding the characterized species, which hinders the establishment of a predictive therapeutic index for the gut microbiota. Meta-analytical data suggest a more consistent relationship between microbial gene content and responsiveness to ICIs compared to microbiome composition. Therefore, further functional studies and clinical trials are warranted to explore the translational implications of these findings.

## Improving gut microbiota efficacy

### Antibiotics

Dysbiosis may contribute to the intestinal inflammation and reduced efficacy of ICIs observed with antibiotic therapy. In experimental colitis, different antibiotic regimens alter the gut microbiota’s ability to control intestinal inflammation by changing microbial community structure and metabolite profiles (152). Streptomycin- and vancomycin-treated microbiota failed to control inflammation, showing proliferation of pathogenic bacteria linked to IBD and metabolites associated with oxidative stress and monosaccharide metabolism. Conversely, metronidazole-treated microbiota maintained inflammation control, with enrichment of Lactobacillus and innate immune responses involving iNKT cells (152). Broad-spectrum antibiotics, and chronic overexposure in the presence of definite or latent infections, can dysregulate the gut microbiota and suppress the immune response. Routy et al. found that in patients receiving PD-1 immunotherapy for epithelial neoplasms, the use of antibiotics (β-lactams+/-inhibitors, fluoroquinolones, or macrolides) led to significantly shorter PFS and OS (12). This indicates that antibiotic therapy, even prior to ICI treatment, can impact treatment efficacy. PINATO et al. similarly reported that prior antibiotic therapy, rather than concurrent therapy, was associated with poorer treatment response and overall survival in patients receiving ICIs (153). Among patients with advanced RCC and NSCLC treated with ICIs, those receiving antibiotics within 30 days of ICI initiation had reduced clinical activity of ICIs compared to those who did not (154). This negative effect of antibiotics on ICIs was also observed in melanoma patients. Patients with advanced melanoma receiving systemic antibiotics along with eplerenone had shorter median overall survival [6.3 m vs. 15.4m, HR=1.88, 95% CI (1.46; 2.43), *P*=10^-6^] (155). Additionally, one study found an increased risk of esophageal, gastric, and pancreatic cancers with increasing penicillin regimens (156). However, some studies suggest a beneficial effect of antibiotics on expanding VδVγ9 T cells in HCC immunotherapy. The efficacy of γδ T cells in tumor therapy in mice models was enhanced by antibiotic-induced gut microbiota dysbiosis (157). Sethi et al. investigated the effect of oral antibiotic depletion of the gut microbiota on tumor growth in models of subcutaneous and hepatic metastases of PDAC, CRC, and melanoma. They found that depletion of the gut microbiota reduced tumor burden in all tested models (158).

Overall, the use of antibiotics during or before the treatment of ICIs is fraught with controversy and is likely to alter the dynamic balance between probiotics and harmful bacteria in the gut. As mentioned above, the use of antibiotics may provide benefits in the treatment of ICIs, but usually, the widespread use of most antibiotics may lead to the development of resistance. Clinicians should consider the duration of antibiotic use to enhance the efficacy of the gut microbiota for ICIs.

### Dietary intervention

The role of diet and dietary interventions in shaping the gut microbiota has garnered significant attention in recent decades. A one-year dietary intervention study conducted in five European countries revealed that adherence to the Mediterranean diet led to alterations in the gut microbiota composition, resulting in reduced frailty and improved health outcomes among older adults (159). This dietary intervention was associated with an increase in the abundance of microbial genes and improvements in clinical phenotypes (160). In another study involving 128 melanoma patients, dietary fiber intake was significantly associated with improved progression-free survival (161). A randomized controlled trial (NCT03275662) demonstrated that a high-fiber diet increased the activity of microbial-encoded glycan-degrading carbohydrate-activating enzymes (CAZymes), leading to a gradual enhancement of microbial diversity, reduced inflammatory markers, and modulation of the immune status (162). Additionally, Nie et al. observed selective increases in the activity of *Mycobacterium avium* spp., *Prevotella* spp., and *Spirochaetes militaris* following consumption of different dietary fiber beverages, along with reductions in the abundance of *Clostridium perfringens* and *Anaplasma fragilis (* 163). Changes in dietary patterns, such as increased consumption of high-fat and high-salt foods, have been implicated in altering the gut microbiota composition and function, thereby promoting chronic inflammation and tumorigenesis. Studies using the azoxymethane (AOM) and APC^min/+^ models of CRC have demonstrated that a high-fat diet can drive CRC development by inducing dysregulation of the gut microbiota, metabolomic alterations including elevated lysophosphatidic acid levels, and dysfunction of the intestinal barrier (164). Similarly, a mice model fed with a high-salt diet showed alterations in the composition and function of the fecal microbiota, reduced butyrate production by Lactobacillus, and disruption of intestinal immune homeostasis through cytokine modulation (165).

### Fecal microbiota transplantation

FMT was initially utilized as a treatment for recurrent *Clostridioides* difficile infections (166). FMT involves the transplantation of gut microbiota from a donor to a recipient. Patients with sustained remission after FMT treatment showed increased alpha diversity, higher abundance of *Lactococcaceae* and *Lactobacillaceae*, reduced levels of *Enterobacteriaceae*, more successful engraftment of donor microbiota, and restoration of gut microbiota ecological balance, compared to non-responders (167). A trial (NCT03568734) assessed the restoration of gut microbiota in infants born via cesarean section following postnatal oral administration of FMT (168). Furthermore, FMT has shown promise in overcoming resistance to ICIs in refractory melanoma (**Table 1**). FMT, when combined with PD-1 inhibitors, has been effective in overcoming PD-1 resistance in refractory melanoma patients. Studies have demonstrated that FMT, in conjunction with PD-1 inhibitors, leads to expansion of activated CD56^+^CD8^+^ T cells, increased activation of CD8^+^ T cells and mucosal-associated invariant T (MAIT) cells in peripheral blood mononuclear cells (PBMCs) in responsive patients (169). Additionally, FMT combined with anti-PD-1 therapy counteracted bone marrow-induced immunosuppression, thereby enhancing CD8^+^ T cell activation in the TME of responsive patients (169). In a multicenter phase I trial (NCT03772899), post-FMT, patients in the responsive group showed immunogenic enrichment and reduction in harmful bacteria (170). Moreover, FMT has shown efficacy in treating immune-related adverse events induced by ICIs, such as ICIs-induced diarrhea and colitis (IMDC). Comparison of pre- and post-treatment fecal samples in patients with complete responses revealed increased α-diversity of the intestinal microbiota and higher abundance of *Cyanobacteria* and *Bifidobacteria* following FMT treatment (171).

**Table 1:** FMT-related ICIs treatment trial.

NCT number	Cancer	Age	Intervention/Treatment	Primary Outcome Measure	Secondary Outcome Measure
NCT04130763	Digestive system cancers	18 - 70	FMT Capsule in Combination with Anti-PD-1 Therapy	ORR; Rate of abnormal vital signs and laboratory test results; The number of adverse events	Change in T-cells Composition; Change in subsets of specific immune system; Change in subsets of non-specific immune system; Function of T-cells;
NCT05750030	HCC	>18	FMT combined with Atezolizumab +Bevacizumab	Safety of atezolizumab/bevacizumab in combination with FMT	CR、PR、SD、PD; ORR、DCR; PFS、OS; Quality of life
NCT03353402	Melanoma	>18	FMT in metastatic melanoma patients who failed immunotherapy	FMT-related adverse events; Proper implant engraftment	Changes in composition of immune cell population; Changes in activity of immune cells
NCT05251389	Melanoma	>18	FMT of an ICI responding or FMT on from an ICI non-responding donor, in combination with ICI.	SD、PR、CR	PFS; The change in gut microbiome following FMT and the duration and stability over time; The immune changes
NCT04056026	Mesothelioma	Child, Adult, Older Adult	A Single Dose FMT Infusion From a Healthy Family Donor Via Colonoscopy as an Adjunct to Keytruda for the Benefit of Improving Efficacy of Immunotherapy for Metastatic Mesothelioma	PFS	/
NCT05690048	Liver Cancer	>18	Vancomycin + Atezolizumab + Bevacizumab + FMT	Differential tumoral CD8 T-cell infiltration;Adverse events	PFS; OS; Change of Hepatic function
NCT04264975	Solid Tumor	>19	FMT in patients who have advanced solid cancer with primary (group 1) or secondary resistance (group 2) to immuno-oncology	ORR	/
NCT05502913	Lung Cancer	>18	Immuno-Oncology Chemotherapy +FMT	PFS	OS; ORR; DCR; Microbiome analysis; Serum antibody levels and lymphocyte subpopulation distribution; Safety and feasibility
NCT04729322	Colorectal Cancer	>18	FMT and Re-introduction of Anti-PD-1 Therapy (Pembrolizumab or Nivolumab) for the Treatment of Metastatic Colorectal Cancer in Anti-PD-1 Non-responders	ORR	/

“/” indicates that the study was not conducted.

Despite the promising results of FMT therapy in patients treated with ICIs, the long-term safety of FMT remains a concern due to its relatively recent adoption as atreatment modality. Two patients in separate clinical trials developed bacteremia caused by broad-spectrum β-lactamase (ESBL) Escherichia coli after receiving FMT from the same donor, resulting in one fatality (172). This severe outcome prompted the U.S. Food and Drug Administration to issue a safety bulletin advising caution and emphasizing the need to avoid FMT from donors carrying potentially pathogenic bacteria. Additionally, a retrospective cohort study revealed that healthy fecal donors could be colonized with multidrug-resistant organisms during donation campaigns. Among 66 donors, 6 (9%) tested positive for multidrug-resistant organisms, with 11 (17%) showing positivity at any given time (173). Regular screening of donor feces is essential to mitigate the risk of transmitting microorganisms that may cause adverse infectious events, particularly in immunocompromised patients. Further clinical investigations are necessary to elucidate the optimal donor selection criteria, transplantation protocols, and recipient characteristics that are critical for the successful implementation of ICI-FMT combination therapy.

### Probiotics and prebiotics

Probiotics, defined as ‘live microorganisms that, when administered in adequate amounts, confer a health benefit on the host (174), have shown promise in CRC treatment. A randomized controlled trial investigating CRC and probiotics revealed that probiotic supplementation improved patients’ quality of life, increased gut microbiota diversity, reduced postoperative infectious complications, and suppressed the production of pro-inflammatory factors (175). Moreover, specific probiotic strains have been found to accumulate at CRC lesion sites after oral administration, where they are fermented by Bacillus butyricus, resulting in the production of SCFAs with known anticancer properties (176). Additionally, probiotic spore-dextrose has been shown to modulate the gut microbiota, enhancing the population of SCFA-producing bacteria and overall microbiota abundance (176). Probiotics have also demonstrated relevance in immunotherapy (**Table 2**). Griffin et al. demonstrated in a murine tumor model that active *Enterococcus faecalis* expresses and secretes a direct homolog of the NlpC/p60 peptidoglycan hydrolase, SagA, leading to the production of immunologically active microparticles (177). Another pro-inflammatory strain, *Bifidobacterium animalis* lactis EDP1503, has shown promise as a novel anti-tumor immunotherapy. EDP1503 reduces the activation of immunosuppressive cells such as myeloid-derived suppressor cells (MDSCs) and regulatory T cells (Tregs), while increasing the number of activated, immune-stimulating DCs, IFN-γ-producing tumor-infiltrating lymphocytes, and cytotoxic CD8^+^ T cells. These effects contribute to the immunogenic remodeling of the TME (178).

**Table 2:** Clinical trial of probiotics and prebiotic-related ICIs.

NCT number	Cancer	Age	Intervention/Treatment	Primary Outcome Measure	Secondary Outcome Measure
NCT05220124	Urothelial Bladder Carcinoma	>18	Live Combined (Bifidobacterium, Lactobacillus and Enterococcus Capsules)	PFS	DOR; OS; SAE; ORR; DCR
NCT04699721	NSCLC	>18	Drug: nivolumab 4.5mg/kg+Paclitaxel (albumin-bound type) 260mg/m2+ Carboplatin AUC5	Adverse Effects; Surgical complications (intraoperative and perioperative); non-R0 surgical events	ORR; Major Pathologic Response; Disease free survival; OS
NCT05032014	Liver Cancer	18–70	Drug: The oral probiotic (Lactobacillus rhamnosus Probio-M9,one times a day during the whole treatment)	ORR	PFS; OS
NCT03817125	Melanoma	>18	Drug: Placebo for antibiotic Placebo for antibiotic will be administered orally four times a day for 4 days, followed by a 2–3 day washout.	Adverse Events	ORR; DCR; PFS; OS; Duration of response
NCT03829111	Kidney Cancer	>18	Drug: Clostridium butyricum CBM 588 Probiotic Strain+ Ipilimumab+ Nivolumab	Change in Bifidobacterium composition of stool	Change in Shannon index; Best overall response; PFS
NCT04009122	NSCLC	>18	IGEN-0206 a sachet after each meal, preferably (3 sachets per day)	Impact of IGEN-0206 on quality of life in patients with metastatic lung cancer according to the EORTC QLQ-C30 Impact of IGEN-0206 on quality of life in patients with metastatic lung cancer according to the EORTC QLQ-L13	BMI; Changes in the microbiota; Interleukin levels; Cytokines levels

Prebiotics, including oligofructose, oligogalactose, and inulin, are known to contain specific chemicals that selectively stimulate the growth of particular bacterial taxa and modify SCFA levels in the gut (179). These substances play a crucial role in maintaining a healthy gut microbiota (**Table 2**). For example, a randomized, double-masked, crossover intervention study (NCT02548247) demonstrated that inulin-type fructans, a type of prebiotic, induced changes in the human intestinal microbiota, particularly affecting the relative abundance of anaerobic digestive bacteria and bifidobacteria (180). In mice with cefixime-induced alterations in the gut microbiota, administration of a prebiotic mixture increased the abundance of Parabacteroides Goldstein and reduced the abundance of *Robinsoniella* psoriasis and *A.muciniphila*. Furthermore, the prebiotic mixture modulated the levels of microbial metabolites, including unsaturated fatty acids and bile acids (181). Studies have also shown that prebiotic oligogalactans can attenuate acute graft-versus-host disease in mice (182). The antitumor effects of inulin have been linked to the activation of intestinal and tumor-infiltrating γδ T cells, which are critical for αβ T cell activation and subsequent control of tumor growth (183). Moreover, adding

inulin or mucin to the diet of C57BL/6 mice has been found to induce antitumor immune responses and inhibit the growth of BRAF mutant melanoma in a subcutaneously implanted mice model (184).

Novel probiotics and prebiotics are being isolated and characterized using various advanced tools, such as sequencing technologies and analytical pipelines. These tools have enabled the identification of new bacterial taxa associated with therapeutic responses. However, there is only modest overlap in the bacterial taxa identified in different studies. While single probiotic strains have shown efficacy in enhancing the effects of ICIs, a combination of multiple strains (flora) may be more effective in maintaining the ecological balance within the gut microbiota. For example, a consortium of 11 species has been shown to confer sustained resistance to Listeria monocytogenes infection in GF mice and to have an additive antitumor effect when combined with anti-PD-1 therapy (103). However, further trials are needed to validate these findings.

### Engineered bacteria

Tumor-targeted engineered probiotics have shown promise in the treatment of CRC. Synthetic probiotics have been engineered to significantly reduce CRC tumor size and inhibit tumor growth. These synthetic probiotics have also been shown to modulate the intestinal microbiota and attenuate chemically induced dysbiosis (185). Tang et al. also demonstrated the inhibition of CRC and modulation of mice intestinal microbial homeostasis, providing further support for this approach (186). Engineered bacteria have been developed to induce systemic antitumor immunity. Chowdhury et al. engineered a nonspecific E. coli strain to specifically lyse and release a nano-antagonist encoding CD47 (CD47nb) in the TME. Topical injection of CD47nb-expressing bacteria stimulated a systemic tumor-antigen-specific immune response and reduced the growth of untreated tumors (187). Another engineered bacterium, SYNB1891, targets STING to activate phagocytic antigen-presenting cells (APCs) in tumors and activate complementary innate immune pathways (188). Additionally, the probiotic E. coli Nissle1917 strain has been shown to colonize tumors and continuously convert ammonia to L-arginine. Increasing L-arginine levels has been associated with increased numbers of tumor-infiltrating T cells and significant synergistic effects in tumor clearance (189). In the CT26 model of microsatellite stable (MSS) CRC in mice, triple-engineered E. coli expressing PD-L1 and anti-CTLA antibodies, as well as granulocyte-macrophage colony-stimulating factor, were found to reduce tumor growth (190).

Engineered bacteria still have limitations. Firstly, they are complex and active agents, rather than precise ones. This complexity can lead to instability in the targeting effect of the bacteria, resulting in varying treatment efficacy among patients with different tumors. Additionally, engineered bacteria may exhibit different levels of immune expression. Low expression levels may result in poor efficacy, while high expression levels may increase the risk of autoimmune diseases. Furthermore, designing personalized treatments with engineered bacteria for different types and stages of cancer requires significant time and economic support, which hinders their widespread use.

## Conclusion

Over the past decade, significant progress has been made in understanding the role of gut microbiota in the typical host environment and diseases. It is now well-established that disruptions in the intestinal microflora can impact the development of various host diseases. The gastrointestinal tract serves as the primary habitat for the intestinal microflora, and its composition and metabolites are closely associated with the occurrence of digestive tract tumors. Research data indicate that the intestinal microflora interacts with the host’s colon epithelium and immune cells through a range of metabolites, proteins, and macromolecules, which regulate the progression of CRC. However, our understanding of the pathogenic mechanisms of the gut microbiota in tumor development and its potential role in immunotherapy remains limited. As a result, numerous unanswered questions in this field still require further investigation.

In recent years, various novel treatment modalities targeting the intestinal microflora have emerged, such as FMT, probiotics, prebiotics, phage therapy, and engineered bacteria, which have significantly advanced our understanding of the role of microbiota in host health. Numerous preclinical and clinical trials investigating the microbiota are currently in progress. Moreover, the abundance and composition of the gut microbiota undergo substantial changes during ICI treatment. Most microorganisms and their metabolites interact with T cells within the immune system. Consequently, the gut microbiota can serve as biomarkers for predicting the efficacy of ICIs.

## Author contributions

YX: Writing – original draft. FL: Writing – review & editing.
